# The ECSIT Mediated Toll3-Dorsal-ALFs Pathway Inhibits Bacterial Amplification in Kuruma Shrimp

**DOI:** 10.3389/fimmu.2022.807326

**Published:** 2022-01-31

**Authors:** Ding Ding, Xue-jun Sun, Meng Yan, Qi Chen, Lin Gao, Cui-jie Kang

**Affiliations:** Shandong Provincial Key Laboratory of Animal Cells and Developmental Biology, School of Life Sciences, Shandong University, Qingdao, China

**Keywords:** Toll, Ecsit, Dorsal, antimicrobial peptides (AMP), *Staphylococcus aureus*, *Vibrio anguillarum*, anti-lipopolysaccharide factor (Alf)

## Abstract

The Toll signaling pathway plays an important role in animal innate immunity. However, its activation and signal transmission greatly differ across species and need to be investigated. Shrimp farming is a worldwide economic activity affected by bacterial disease from the 1990s, which promoted research on shrimp immunity. In this study, we first proved that, among the three identified Toll receptors in *Marsupenaeus japonicus* kuruma shrimp, Toll 3 plays a pivotal role in initiating the antibacterial response *in vivo*, especially upon anti-*Staphylococcus aureus* infection. Further research showed that this result was due to the activation of the Dorsal transcription factor, which induced the expression of two anti-lipopolysaccharide factors (Alfs). Moreover, the evolutionarily conserved signaling intermediate in Toll pathways, ECSIT, was proved to be needed for signal transmission from Toll 3 to Dorsal and the expression of anti-lipopolysaccharide factors. Finally, the mortality assay showed that a Toll3-ECSIT-Dorsal-Alf axis was functional in the anti-*S.aureus* immunity of *M. japonicus* shrimp. The results provide new insights into the function and signal transduction of the Toll pathway in aquatic species and offer basic knowledge for shrimp disease control and genetic breeding.

## Introduction

Innate immunity is a basic first defense mechanism of multicellular organisms. It is generally initiated by recognizing pathogen-associated molecular patterns (PAMPs) of invading organisms *via* multiple membrane-located pattern recognition receptors (PRRs) on the host cell (1, 2). Among PRR members, the Toll (invertebrate) or Toll-like receptor (TLR) (vertebrate) superfamily is the most important and multifunctional PRR family member (3–5). To date, the activation and transduction of the Toll/TLR signaling pathway in mammals and fruit fly species have been well illustrated, namely, the activation of Toll/TLR by specific ligands, recruitment of downstream signaling molecules, activation of transcription factor translocation, and induction of effector molecule production (6, 7). Studies have shown that the basic function and components of Toll/TLR signaling are similar across various species, unlike the differing activation and transduction of immune signals downstream of them. For instance, the classical Toll pathway in *Drosophila* responds to external infection with Gram-positive bacteria and fungi and oral infection with several RNA viruses. The activation of Toll receptors needs a series of proteinase cascade reactions and cleavage of the Spätzle ligand. After the phosphorylation and degradation of the inhibitor Cactus (mammalian IκB homologue), the transcription factor Dorsal or the Dorsal-related immunity factor (Dif) is released and translocated to the nucleus to induce the expression of antimicrobial peptides (AMPs) (8, 9). By contrast, several TLRs in humans could recognize a broad range of PAMPs directly. Then, additional signaling adaptor proteins, such as TAK/TAB and IKKs (which are absent from the *Drosophila* Toll signaling pathway but present in its imd signaling pathway) are recruited for signal transduction. Additionally, more than one transcription factor (namely, nuclear factor-κB [NF-κB], interferon-regulatory factors [IRFs], cyclic AMP-responsive element-binding protein [CREB], or activator protein 1 [AP1]) are activated downstream of different TLR receptors and trigger the production of pro-inflammatory cytokines or Type I IFNs (IFNα and IFNβ). Besides that, while the function of all human TLRs is activating immune signal transduction, only five *Drosophila* Toll receptors (Toll, Toll2, Toll5, Toll7, and Toll9) among the nine identified, have been implicated in the fruit fly immune response thus far (10).

Research on the shrimp innate immune system has been drawing extensive interest since the 1990s, because of the economic value of the animal and the need for disease control strategies (11–15). To date, dozens of Toll receptors have been identified in various shrimp species, namely, three *Mj*Tolls in *Marsupenaeus japonicas* (16), nine *Lv*Tolls in *Litopenaeus vannamei* (17), one *Fc*Toll in *Fenneropenaeus chinensis* (18), two *Pm*Tolls in *Penaeus monodon* (19, 20), five *Mr*Tolls in *Macrobrachium rosenbergi*, and six *Pc*Tolls in *Procambarus clarkii* (21). Besides that, some key components involved in classic Toll pathway signal transduction were also identified in shrimp (namely, Dorsal, Cactus, Spätzle, MyD88, Tube, Pelle, TRAF6, etc.), highlighting the conservation of the Toll signaling pathway in aquatic arthropods (22–29). To date, the shrimp Tolls have been shown to respond to bacterial or viral infection and lead to Dorsal activation and the expression of AMPs in several shrimp species. However, only two identified Toll signaling pathways have been reported, the *Pc*Toll2-ATF4-ALF1/2 pathway in *P. clarkii* exposed to *Vibrio parahemolyticus* and the *Lv*Toll4-Dorsal- ALF1/LYZ1 pathway in *L. vannamei* responding to white spot syndrome virus infection (17, 30). The key Toll receptor responses to different pathogens and the downstream signal transduction pathways regulating effector genes are still largely unknown in other shrimp species.

An evolutionarily conserved signaling intermediate in Toll pathways, known as ECSIT, was initially cloned as a tumor necrosis factor receptor-associated factor 6- (TRAF6-) interacting protein by yeast two-hybrid screening in mice. Through interactions with TRAF6 and MEKK1, ECSIT offers alternative means to activate NF-κB and AP-1 in mammalian TLR4 signaling (31). The interaction of *Dm*ECSIT and *Dm*TRAF6 protein is also conserved in *Drosophila*, and *DmEcsit* efficiently activates AMP expression in S2 cells (31). However, genetic research has shown that *Drosophila* TRAF homologues do not participate in immune signal transduction, and the *in vivo* immune function of *DmEcsit* in *Drosophila* was also not reported (32). In our previous research, a shrimp *ECSIT* homologue was cloned from *M. japonicus*, named *MjEcsit1*. Quantitative real-time polymerase chain reaction (qRT-PCR) analysis showed detected its transcription in all test tissues and its upregulation upon *Vibrio anguillarum* or *Staphylococcus aureus* infection. Through RNA interference combined with survival and bacterial clearance assays, we showed that the shrimp *ECSIT* gene functioned in the anti-*S. aureus* immune response by regulating the expression of several AMPs (26). However, among the three identified *Mj*Tolls, the key receptor responding to bacterial infection remains unknown, as does whether and how shrimp ECSIT participates in Toll signaling transduction.

In the present study, an RNAi screen and bacterial clearance assay were used to identify the function of three Tolls in the antibacterial immunity of shrimp *M. japonicas*.

## Materials and Methods

### Immune Challenge, Sample Collection and Preparation

*M. japonicus* kuruma shrimps (approximately 8–10 g each) were obtained from a local seafood market in Jinan, Shandong Province, China. They were cultured in tanks with air pumps and circulating seawater at 22 °C. The shrimps were divided into experiment and control groups for an immune challenge, with 20 individuals in each group. In the experimental group, 10 μl of *V. anguillarum* or *S. aureus* suspension (3 × 10^8^ CFU) were injected into the last abdominal segment of each shrimp. The same volume of phosphate-buffered saline (PBS: 10 mM Na_2_HPO_4_, KH_2_PO_4_, 137 mM NaCl, 2.7 mM KCl, and 10 mM MgCl_2_, pH 7.4) was injected into the control. At different times post-infection, shrimp hemolymph was collected with equal volumes of anticoagulant agent (450 mM NaCl, 10 mM KCl, 10 mM EDTA, and 10 mM HEPES, pH 7.45) and centrifuged at 800 ×*g* for 7 min at 4°C to obtain the hemocyte pellet. Other tissues (gills, hepatopancreas, stomach, heart, and intestine) were collected simultaneously. Each sample originated from at least four shrimps. Total RNA was extracted using a Unizol reagent (Biostar Pharmaceuticals, Inc., Shanghai, China) from about 100 mg of tissue or 3 × 10^7^ cells, and the cDNA was synthesized using a FastQuant First Strand cDNA Synthesis kit (TIANGEN, Beijing, China) according to manufacturer’s instructions.

### Recombinant Expression and Antibody Preparation

The nucleotide fragment encoding the ECSIT domain of MjEcsit1 and beta-actin gene was amplified using the primers ecsitexF/ecsitexR or actinexF/actinexR (**Table 1**). Fragments were then ligated into the *Bam*HI and *Xho*I restriction enzyme sites of the pET-30a(+) vector. The recombinant plasmid was transformed into competent *Escherichia coli* BL21(DE3) cells. Recombinant protein expression was induced by 0.5 mM isopropyl thiogalactoside (IPTG) at 28°C for 6 h. ECSIT domain product was insoluble and purified using His•Bind resin chromatography (Novagen, USA) after refolding by three-step dialysis. The purified protein was separated by 12.5% SDS-PAGE staining by Coomassie Blue. Protein concentration was assessed by the Bradford method (33).

**Table 1:** Sequences of primers in this study. Restriction enzyme cutting sites are underlined. T7 promoter sequence in primers was italicized.

Primers	Primer Sequences ( 5’-3’)
**Q-PCR analysis**	
*ecsit*rtF	TTTATTTATGCTGCTCTTAGGC
*ecsit*rtR	ATCATCTCCATCTCTGTATCT
*alf*1rtF	CAAAGTTGTTGGGTTGTGGA
*alf*1rtR	CGGACTGGCTGCGTGTG
*alf*3rtF	CTCTACAGCAACGGCACA
*alf*3rtR	ACACCACATCCGACCCT
*alf*5rtF	CTGGTCGGTTTCCTGGTGGC
*alf*5rtR	TTGGGTTGTGGCACTCGG
*alf*6rtF	TGGTGGTGGCAGTGGCT
*alf*6rtR	CGGGTCTCGGCTTCTCCT
*alf*8rtF	CGCAGGCTTATGGAGGAC
*alf*8rtR	GGTGACAGTGCCGAGGA
*crustin*4rtF	CTCCACCACTCTCGCACTAACA
*crustin*4rtR	TGATGGTCTCAGATTGGGGC
*crustin*11rtF	TTTTCGTCTTCGTCCTGATGG
*crustin*11rtR	ATTGTAGTCCTTTCCGCCGTC
*β*-*actin*rtF	CAGCCTTCCTTCCTGGGTATGG
*β*-*actin*rtR	GAGGGAGCGAGGGCAGTGATT
*toll*1rtF	TGTGCCCCATCCTTCTGC
*toll*1rtR	ACCACAGCCCACAAGCACA
*toll*2rtF	CCATAACAGAGGACGAATTAGAT
*toll*2rtR	TAGTGGAGGCAAATGCGGTA
*toll*3rtF	GAGGCACTGCGAGGGAA
*toll*3rtR	GAGACGTGGCTGAGGTATGG
*Traf6rtF*	AACTAAACCAGGTCTTCAGGCTT
*Traf6rtR*	CTTTTCCGTGCTTTGATTATTCT
**RNAi**	
*ecsit*iF	*GCGTAATACGACTCACTATAGG*GGGCTGCCTTTCAGTGTGC
*ecsit*iR	*GCGTAATACGACTCACTATAGG*GATATGTAGTGATTTTTGATGTCG
*toll*1iF	*GCGTAATACGACTCACTATAGG*GCCATCCTTCTGCCACCTAA
*toll*1iR	*GCGTAATACGACTCACTATAGG*GAATCTGATTTGACAAGTTCCA
*toll*2iF	*GCGTAATACGACTCACTATAGG*GTAAAGTCCTTGATGTGCGAG
*toll*2iR	*GCGTAATACGACTCACTATAGG*GTGTATAAGTTCTTGTGGGTGT
*toll*3iF	*GCGTAATACGACTCACTATAGG*GTGGAGCGTGGAGACAGGCCC
*toll*3iR	*GCGTAATACGACTCACTATAGG*GGCTGTTGACACTGTACTTGT
*GFP*iF	*GCGTAATACGACTCACTATAGG*GTGGTCCCAATTCTCGTGGAAC
*GFP*iR	*GCGTAATACGACTCACTATAGG*GCTTGAAGTTGACCTTGATGCC
*alf*5iF	*GCGTAATACGACTCACTATAGG*GAGCATCGCATACGGACAT
*alf*5iR	*GCGTAATACGACTCACTATAGG*GCTCGGTGATAAGGTTTCTT
*alf*6iF	*GCGTAATACGACTCACTATAGG*GCATGATCCTGGTGGTGGC
*alf*6iR	*GCGTAATACGACTCACTATAGG*GTGCGGGTCTCGGCTTCT
**Recombinant expression**	
*ecsit*exF	TATC GGATCC GCAAATCCTCAACACAC
*ecsit*exR	TATC CTCGAG CTAGCCACTAATAATCC
*alf*5exF	TATC GGATCC ATGCGTTTCCTGGTCGG
*alf*5exR	TATC GGATCC TCAATCTTCCAGCCAG
*alf*6exF	TATC GGATCC ATGCGAGTGTCGCTAC
*alf*6exR	TATC GGATCC TTACTGATTTAACCAAG
*dorsal*exF	TACT CAGGAT CCGACCCTGATCTGGAGAGT
*dorsal*exF	TACT CACTCG AGGTACTGGGGATCTGAGTC
*actin* exF	TACT CAGGAT CCATGTGCGACGAGGAAGTT
*actin* exR	TACT CACTCG AGGGAGGTGGAGGCGGCAGC

For the recombinant expression of shrimp Dorsal, Alf5, and Alf6, the nucleotide fragments were amplified with primer pairs *dorsal*exF/*dorsal*exR, *alf*5exF/*alf*5exR, and *alf*6exF/*alf*6exR, respectively (**Table 1**), and ligated into the *Eco*RI and *Xho*I sites of the pET-32a(+) vector. Protein expression was induced in *E. coli* Rosetta (DE3) cells by adding 0.5 mM IPTG at 37°C for 5 h. Recombinant proteins were purified by His•Bind resin chromatography (Novagen, USA), according to the manufacturer’s instructions.

Antisera for shrimp ECSIT, Actin, and Dorsal proteins were prepared by the Qingdao Kangda Biotechnology company through using the purified recombination protein of the rECSIT domain, rACTIN, and rDorsal following the method described in a previous research (34), and then frozen at −80 °C for use. Antibody specificity was detected by Western blot (**Supplementary Figure 1**).

### Gene Expression Profile Analysis

Quantitative real time PCR (qRT-PCR) was performed using an Ultra SYBR mixture protocol (with ROX, CWBio, Beijing, China) and C1000 thermal cycler (Bio-Rad, USA) to determine gene expression profiles. Gene-specific primers are listed in **Table 1**. The cycling conditions were: 94°C for 5 min; 40 cycles of 94°C for 10 s, 60°C for 1 min, and a melting curve from 65 to 95°C. *β-actin* was used as the internal reference gene. Expression levels were normalized relative to those of the control group for each time point. The results were analyzed using the 2^−ΔΔCt^ method and GraphPad Prism software (GraphPad, San Diego, CA, USA). Three independent experiments were performed, and data were statistically analyzed using the student’s t-test and presented as the mean ± SD. Significant differences were accepted at *p <*0.05 (*p <0.05, **p <0.01).

Western blot was performed to analyze the *Mj*Ecsit1 tissue distribution and *Mj*Dorsal translocation and expression pattern after bacterial challenge. Tissues (25 mg for each) collected from four individuals were polled together and homogenized in lysis buffer (50 mM Tris–HCl pH 7.5, 150 mM NaCl, 0.1% SDS, 3 mM EDTA) and centrifuged at 14,000×*g* for 15 min at 4 °C to collect the supernatant. The nucleoprotein and cytoplasmic protein were extracted from the shrimp gills following the manufacturer’s instructions of the “Membrane, nuclear, and cytoplasmic protein extraction kit” (Sangon Biotech, Shanghai, China). After determining protein concentration by the Bradford method, 100 μg protein were resolved by 12.5% SDS-PAGE and transferred onto a nitrocellulose membrane. The membrane was blocked with 3% skimmed milk in Tris-buffered saline for 1 h (TBS: 150 mM NaCl, 10 mM Tris–HCl, pH 7.5) and incubated with a specific primary antibody (as prepared in *Recombinant Expression and Antibody Preparation*) at 4°C overnight. After washing three times by TBST (TBS, 0.02% Tween-20), the membrane was incubated with Alkaline Phosphatase Goat anti-Rabbit IgG (H + L) (1:10,000 in blocking buffer, ZSGB Bio, Beijing, China) for 4 h. Finally, the membrane was washed by TBST, and protein bands were developed using a color-developing buffer (10 ml TBS, 45 μl NBT, and 35 μl BCIP). Western blot results were analyzed by Quantity One and GraphPad Prism software.

### Immunocytochemistry Assays

Hemolymph was collected from three shrimps (10 g per shrimp) using a 5 ml syringe preloaded with 1 ml of anticoagulant (0.45 M NaCl, 10 mM KCl, 10 mM EDTA, and 10 mM HEPES, pH 7.45) and then fixed by adding 1 ml 4% paraformaldehyde. Hemocytes were collected by centrifugation at 700 ×*g* for 5 min at 4°C, and then washed with PBS for three times, incubated in 0.2% Triton X-100 at 37°C for 5 min, washed with PBS five times, and then blocked by 2% bovine serum albumin (BSA, dissolved in PBS) at 37°C for 30 min. Then, anti-Dorsal (1:200 in blocking regent) was added and samples were incubated overnight at 4°C. After washing with PBS, the hemocytes were incubated with 2% BSA for 10 min, then the second antibody (goat anti-rabbit-Alexa Fluor 488, 1:1,000 diluted in 3% BSA) was added, and samples were incubated for 1 h at 37°C and washed with PBS. Hemocyte nuclei were stained with 4′,6-diamidino-2-phenylindole dihydrochloride (DAPI, AnaSpec Inc., San Jose, CA) for 10 min at room temperature and washed again. The results were observed under a fluorescence microscope (Olympus BX51, Tokyo, Japan) and ImageJ was used to calculate the colocalization percentage of Dorsal with nuclei stained with DAPI.

### RNA Interference (RNAi)

RNAi was performed through double-stranded RNA (*ds*RNA) injection to detect gene function *in vivo*. The partial DNA fragments of indicated genes and the control gene (*GFP*) were amplified using primers linked to a T7 promoter (**Table 1**). The PCR products were purified, enriched to 1 μg/μl, and utilized as templates for *ds*RNA synthesis. *ds*RNA was synthesized following the instructions stated in a MEGAscript™ T7 transcription kit (AM1334, Thermo Fisher Scientific).

For the RNAi assay, shrimps were divided into the control group (*dsGFP*) and experimental group. Each juvenile shrimp (6–8 g) was injected with *ds*RNA (3 μg/g shrimp) at the last abdominal segment. The *ds*RNA injection was repeated after 24 h. Shrimp gills were collected 24 h after the second *ds*RNA injection. Total RNA was extracted and subjected to cDNA synthesis using a commercial kit (Cat#G492, abmGood, Canada), in accordance to manufacturer’s instructions. The cDNAs were diluted 20-fold in nuclease-free water. The efficient RNAi fragment was selected after three independent experiments and used in the next experiment. The PCR primers for them are shown in **Table 1**, and the RNAi efficiency was detected before each further experiment.

### *In Vivo* Bacterial Clearance Assay

Bacterial clearance assays were performed to determine whether shrimp *Toll* or *Alf* genes participated in inhibiting bacterial proliferation *in vivo*.

Shrimp were divided into the experimental groups (7 individuals in each group) and a control group (*GFP* knockdown + bacterial challenge). Bacteria *V. anguillarum* or *S. aureus* (3 × 10^8^ CFU) was injected into specific gene-silenced or *GFP* gene-silenced shrimp. At 6 h after bacterial injection, 200 μl hemolymph was collected from the ventral sinuses of each shrimp. Then, 10 μl hemolymph was exacted from each sample, diluted 100-fold with sterile PBS, and spread onto a 2216E plate (5% tryptone, 1% yeast extracted, 1.5% agar, 0.01% FeCl_3_, and seawater). Four parallel operations were conducted for each shrimp. The 2216E plates were incubated at 37°C overnight, and bacterial colonies were counted the next day. The average number of colony counts of four parallel quantifications for one shrimp was utilized as the final data, which were entered into GraphPad Prism for analysis. The differences between experimental and control groups (*dsGFP*) were analyzed by the *t-test* and shown based on *p*-values (*p <0.05, ** *p <*0.01).

### Bacterial Binding Assay

Gram-negative (*V. anguillarum* and *E. coli*) and Gram-positive (*S. aureus*, *Bacillus megaterium*, *Bacillus thuringiensis*, and *Bacillus subtilis*) bacteria were selected to assess the binding activity of recombinant ALF5 and ALF6 proteins following an existing method (32). Bacteria were cultured in LB media (1% NaCl, 1% tryptone, and 0.5% yeast extract) and collected in the mid-logarithmic phase by centrifugation at 6,000 rpm for 5 min. After being washed twice and resuspension in TBS (15 mM NaCl, 100 mM Tris–HCl, pH 7.5), bacteria (approximately 2 × 10^6^ cells) were incubated with 100 μl of purified recombinant ALF5 or ALF6 protein (0.8 mg/ml) by shaking at room temperature for 1 h. Subsequently, bacteria were separately washed in a TBS and 7% SDS solution. Pellets were resuspended in 50 μl TBS and analyzed by 15% SDS-PAGE for Western blotting. A mouse anti-histidine monoclonal antibody (ZSGB Bio, Beijing, China) was utilized as the primary antibody (1:3,000), whereas alkaline phosphatase-conjugated horse anti-mouse IgG (H + L) (ZSGB Bio, Beijing, China) was the secondary antibody (1:10,000). Purified recombinant protein TRX was utilized as the negative control to eliminate the effect of tag protein in r*Mj*Alf5 or r*Mj*Alf6.

### Antimicrobial Activity Analysis

Antimicrobial activities of recombinant Alfs were tested by liquid growth inhibition assays as minimum inhibitory concentration (MIC) values. Two Gram-positive bacteria (*S. aureus* and *B. thuringiensis* subsp. Kurstaki) and two Gram-negative bacteria (*E. coli* and *V. anguillarum*) were used in this experiment. Briefly, bacterial cells harvested at the mid-logarithmic phase were diluted to 2 × 10^5^ CFU/ml in Poor Broth (1% tryptone, 0.5% NaCl (w/v), pH 7.5) and 90 µl/well bacteria were added into a 96-well polypropylene microtiter plate. In the test, 10 µl/well of serially diluted recombinant Alf protein or the control protein (bovine serum albumin) were added to the 96-well plate. The final concentration of peptides in the medium ranged from 3.00, 1.50, 0.75, 0.38, 0.19, to 0.09 μM. The mixtures were incubated for 48 h with vigorous shaking at 30°C, and bacterial growth was evaluated by measuring the culture absorbance at 600 nm using a microplate reader. The minimal growth inhibition concentration (MIC) was expressed as the lowest final concentration of the protein at which no bacterial growth was observed compared with that in control. The assay was conducted in twice, with triplicate in each protein concentration.

### Survival Rate Assay

The survival rate assay was performed to confirm the function of *Toll*3 and *Alf*6 *in vivo*. Shrimps were equally divided into three groups (30 individuals in each group), namely, one control group (*dsGFP* + *S. aureus*) and two experimental groups (*dsToll*3 + *S. aureus* and *dsAlf*6 + *S. aureus*). Each shrimp was injected with 3 μg/g *ds*RNA twice. Interference effects were detected by qRT-PCR 24 h after *S. aureus* challenge (1 × 10^8^ cells). The number of dead shrimps was counted every 12 h, and the survival rates of the three groups were calculated. The cumulative survival rates and significant differences between the control and experiment groups were analyzed by GraphPad Prism and are indicated by asterisks (*p <0.05, ** *p <*0.01).

## Results

### RNAi Screening Identifies the *In Vivo* Function of Shrimp Tolls

**Figure 1:**
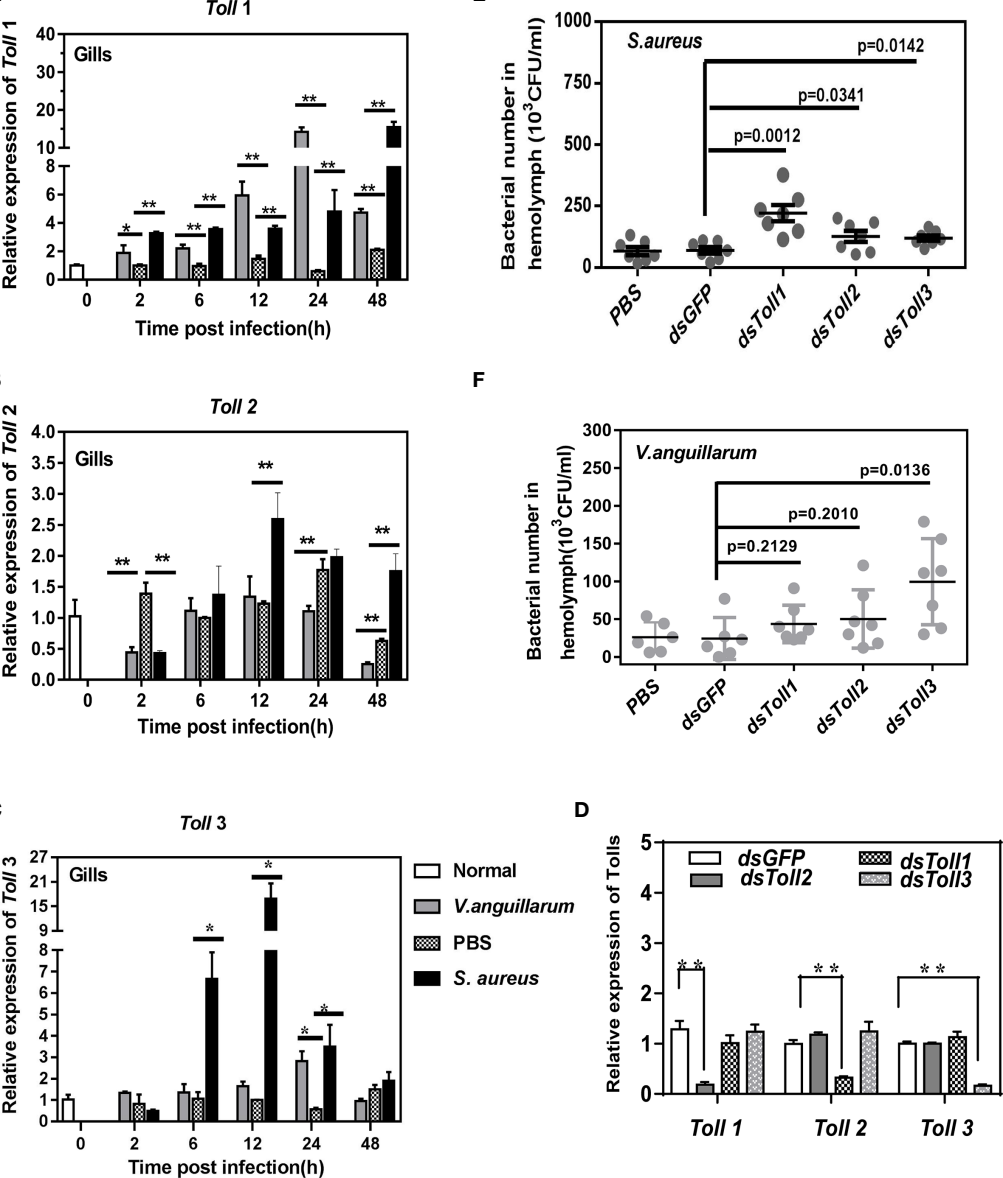
Expression and function of *Toll*s in shrimp. The expression patterns of *Toll*s in shrimp gills upon *V. anguillarum* or *S. aureus* infection were detected by qRT-PCR. **(A–C)** Significant differences were analyzed between the bacteria-challenged samples and the PBS-injected samples by paired *t*-test analysis and were marked by asterisks (*p < 0.05, **p < 0.01). **(D)** The knockdown specificity and efficiency of *Tolls* in shrimp gills were analyzed by Q-PCR. Significant differences were analyzed by paired student’s t-test analysis and are indicated by asterisks (*p <0.05, **p <0.01). Bacterial clearance assay was performed after RNAi to detect the function of *Toll*s in *V. anguillarum* **(E)** or *S. aureus* **(F)** clearance. The differences between *dsTolls* and control groups (*dsGFP* and PBS) were checked by student’s *t*-test and are shown by *p*-values.

Firstly, qRT-PCR was performed to investigate shrimp Toll expression patterns during Gram-negative (*V. anguillarum*) or Gram-positive (*S. aureus)* bacterial infection. The high expression of Tolls in gill tissues, together with the importance of gills tissue in shrimp breath and immunity, made us choose gills as the main organ for further study (13, 22). The transcription of *Toll1* in the gills was noticeably upregulated by two kinds of bacterial challenge at all the tested time points, the transcription of *Toll2* was upregulated only by *S. aureus* infection at a later time point in the gills, and the expression of *Toll3* increased at 6–24 h post-*S. aureus* infection and at 24 h post-*V. anguillarum* infection (**Figures 1A–C**). These findings indicated that three *Toll*s may respond to bacterial infection in shrimp tissues.

To confirm the function of Toll receptors *in vivo*, the bacterial clearance ability of shrimp was detected before and after *Toll1/2/3* knockdown. Firstly, the specificity and efficiency of Toll receptor knockdown was assured and confirmed by qRT-PCR assay (**Figure 1D**). Next, the *V. anguillarum* or *S. aureus* populations in *Toll*-silenced shrimp were counted and compared with those of the control group. As seen in **Figure 1E**, *Toll1/2*-silenced shrimp showed no noticeable differences in the *V. anguillarum* number compared with that of the control group (*dsGFP*-injected shrimp) (*p >*0.05), whereas a significantly higher number of *V. anguillarum* clones was seen in *Toll3*-silenced shrimp (*p* = 0.0136). Therefore, *Toll3* may be the key *Toll* mediating the anti-Gram-negative bacteria response in shrimp.

By contrast, the *S. aureus* numbers in *Toll1/2/3*-silenced shrimp were significantly higher than those in the *dsGFP*-injected shrimp (**Figure 1F**). Thus, *Toll1/2/3* all participated in the anti-Gram-positive bacteria immune response. Therefore, the function of *Toll*s during *S. aureus* infection was compared and investigated.

### The Expressions of Alf5 and Alf6 are Regulated by Toll 3

**Figure 2:**
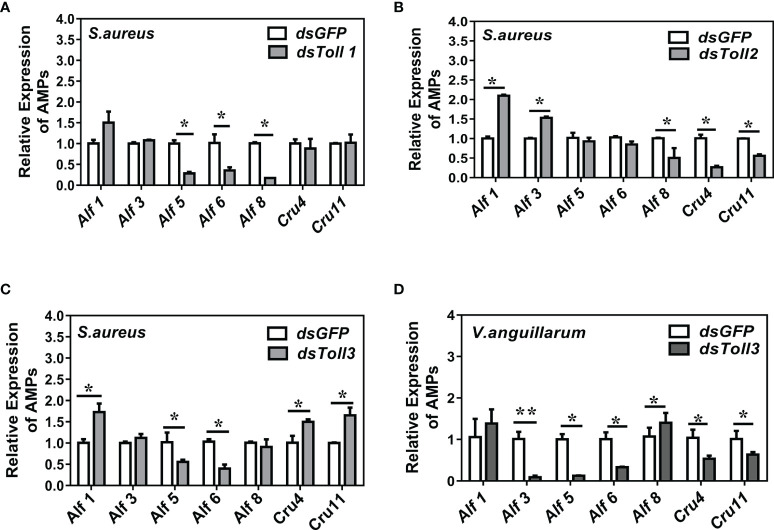
The expression of AMP genes in shrimp under *Toll* knockdowns and bacterial infection. **(A–C)** *Toll*1-3 knockdowns and *S. aureus* infection. **(D)** Toll3 knockdown and *V. anguillarum* infection. Bacteria (~10^8^ CFU) were injected 24 h after *Toll* knockdown. Total RNAs were extracted from shrimp gills 6 h post-bacterial infection, qRT-PCR analysis was performed to test the expression of AMP genes. Three biological repeats were performed. Significant differences between the *dsTolls* and the *dsGFP* samples were analyzed by paired student’s *t*-test analysis and are indicated by asterisks (*p < 0.05, **p < 0.01).

In *S. aureus*-challenged shrimp, compared with the control group (*dsGFP* injection), *Toll1* knockdown inhibited the expression of *Alf4*, *Alf5*, *Alf6*, and *Alf8* (**Figure 2A**). *Toll2* knockdown inhibited the expression of *Alf4*, *Alf8*, and C*rustin4*, but promoted the expression of *Alf3* (**Figure 2B**). In *Toll3*-silenced shrimp, the expression of four AMPs was downregulated (*Alf4*, *Alf5*, *Alf6*, and *Alf8*) and AMPs were upregulated (*Alf1*, *Crustin4*, and *Crustin11*) (**Figure 2C**).

Since *Toll3* also mediated clearance of Gram-negative bacterial *in vivo*, the gene expression patterns of shrimp under *Toll3* knockdown and *V. anguillarum* infection were also detected. Notably, the expression of four AMPs (*Alf3*, *Alf5*, *Alf6*, and *Crustin4*) was inhibited (**Figure 2D**). The expressions of *Alf* 5 and *Alf* 6 were suppressed upon *V. anguillarum* or *S. aureus* challenge and *Toll* 3 silencing, indicating that they were the readout genes downstream of the *Toll* 3 signaling pathway.

### Toll3 Regulates the Expression of Alfs Through Dorsal Activation

**Figure 3:**
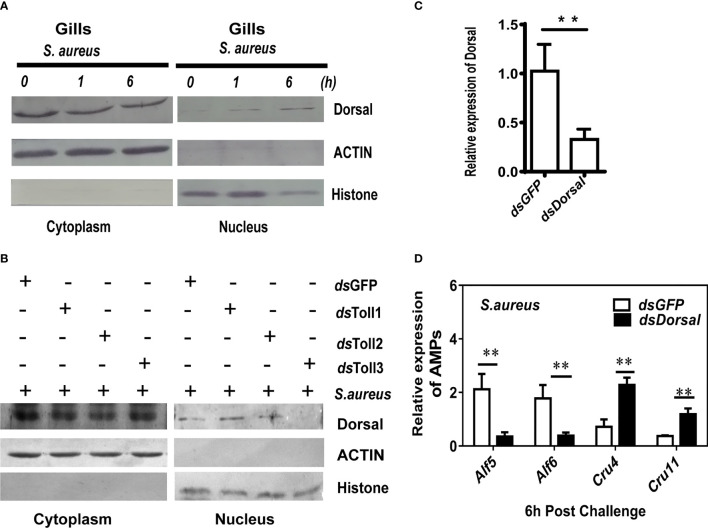
Toll3 regulates the expression of *Alf*s through Dorsal activation. **(A)** Western blot detection of the cellular location of Dorsal 1- and 6-h post-*S. aureus* infection in shrimp gills. **(B)** Western blot detection of the nuclear translocation of Dorsal in *Toll*1-, *Toll*2-, and *Toll*3-silenced shrimp. **(C)** Detection of the RNAi efficiency of Dorsal by qRT-PCR. **(D)** qRT-PCR detection of the expression of AMPs in *Dorsal*-silenced shrimp and the control group. Significant differences between the *dsDorsal* and the control group (*dsGFP* samples) were analyzed by paired student’s *t*-test analysis and are indicated by asterisks (*p < 0.05, **p < 0.01).

**Figure 4:**
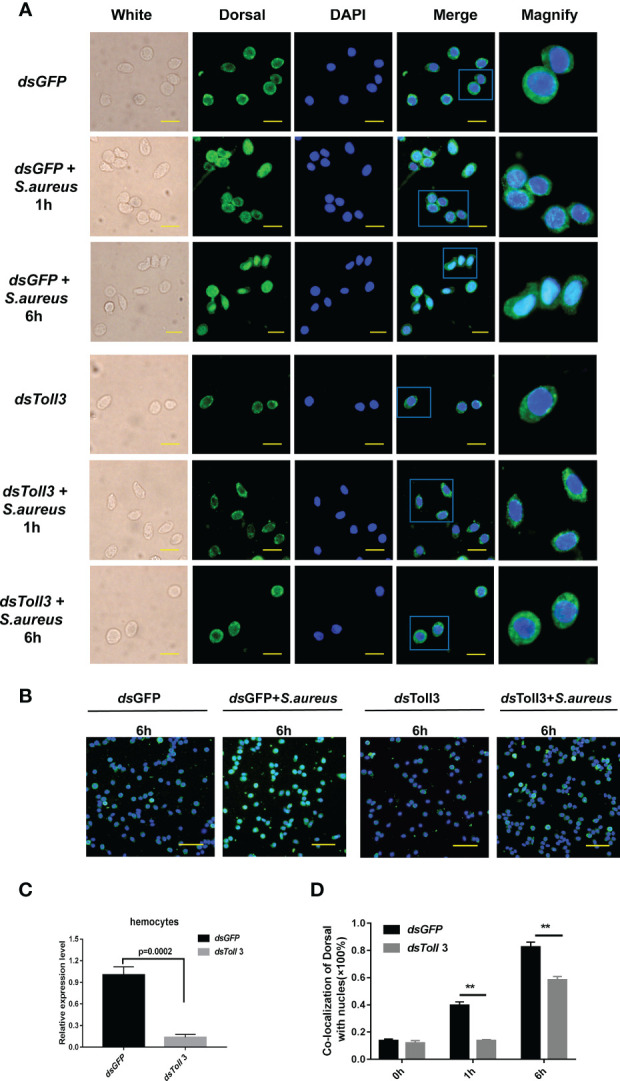
Knockdown of *Toll*3 inhibits the nuclear translocation of Dorsal in shrimp. **(A)** Translocation of Dorsal in hemocytes of *Toll*3-silenced shrimp challenged with *S. aureus* at 1 and 6 h post-infection. Green fluorescence signal indicates the distribution of Dorsal in hemocytes, and blue shows the nucleus stained with DAPI. *dsGFP* was used as control. Bar = 20μm. **(B)** Dorsal translocation in hemocytes of *Toll*3-silenced shrimp 6h after *S.aureu*s challenge compared with that in the *dsGFP* group. Green fluorescence signal indicates the distribution of Dorsal in hemocytes; blue shows the nucleus stained with DAPI. Bar = 30μm. **(C)** Efficiency of RNAi for *Toll*3 in shrimp hemocytes based on RNA levels. *dsGFP* injection was used as a control. **(D)** Statistical analysis of **(A)** *via* Image J software. Significant differences between the *dsToll3* and the control group (*dsGFP* samples) were analyzed by paired student’s t-test analysis and are indicated by asterisks (**p < 0.01).

To clarify whether *Toll3* regulates the expression of *Alf5* and *Alf6* through the classic transcription factor Dorsal, its activation was detected first. Cytoplasmic or nuclear proteins were prepared from the gills of normal shrimp and 1 h or 6 h after challenge with *S. aureus*. Dorsal translocation from the cytoplasm to the nucleus upon *S. aureus* infection was detected using a Dorsal-specific antibody (**Supplementary Figure 1**). Most of the Dorsal signal was located in the cytoplasm under normal conditions, and appeared in the nucleus at 1 h and kept increasing up to 6 h after the *S. aureus* infection, indicating its translocation from the cytoplasm into the nucleus in gills after *S. aureus* challenge (**Figures 3A**, **4A**).Next, the cellular distribution of Dorsal was detected after RNAi of *Toll*s in shrimp at 6 h post-*S. aureus* challenge. Compared with the control group (*dsGFP*-treated), Dorsal signals in the cytoplasm and nucleus of gills were not altered in *Toll1*- and *Toll2*-silenced shrimp challenged with *S. aureus*. In *Toll3-*silenced and *S. aureus*-challenged shrimp, the Dorsal signal was detected only in the cytoplasm of gills, and the signal in the nucleus was not detectable, indicating that nuclear translocation of Dorsal was blocked by *Toll3* interference (**Figure 3B**). Similar results were observed in hemocytes. Most of the Dorsal signal was located in the cytoplasm under normal conditions, it appeared in the nucleus at 1 h and kept increasing until 6 h after the *S. aureus* infection, indicating that Dorsal transferred from the cytoplasm into the nucleus after *S. aureus* challenge (**Figure 4A**). In *Toll3*-silenced shrimp, the nuclear translocation of Dorsal in hemocytes was inhibited compared with that in the control group (**Figures 4B–D**).

To investigate whether the expression of readout genes of *Toll3* was controlled by Dorsal, the Dorsal expression was interfered by *ds*RNA injection, and the expression patterns of *Alf5, Alf6*, and two other genes (*Crustin4* and *Crustin12*) was detected by qRT-PCR. Compared with the control group (*dsGFP* injection), the expressions of *Alf5* and *Alf6* were suppressed in shrimp gills under *Dorsal* knockdown and *S. aureus* infection (**Figure 3D**). Conversely, the expressions of *Crustin4* and *Crustin12* were not challenged under *dsGFP* knockdown and *S. aureus* infection, and were upregulated upon *Dorsal* knockdown and *S. aureus* infection (**Figure 3C**). Taken together, these results suggested that in shrimp gills, *S. aureus* infection activates the Toll3 pathway through facilitate the nucleus translocation of Dorsal and induce the expression of *Alf*5 and *Alf*6.

### Alf5 and Alf6 Are Efficient in Anti-*S. aureus* Infection in Shrimp

**Figure 5:**
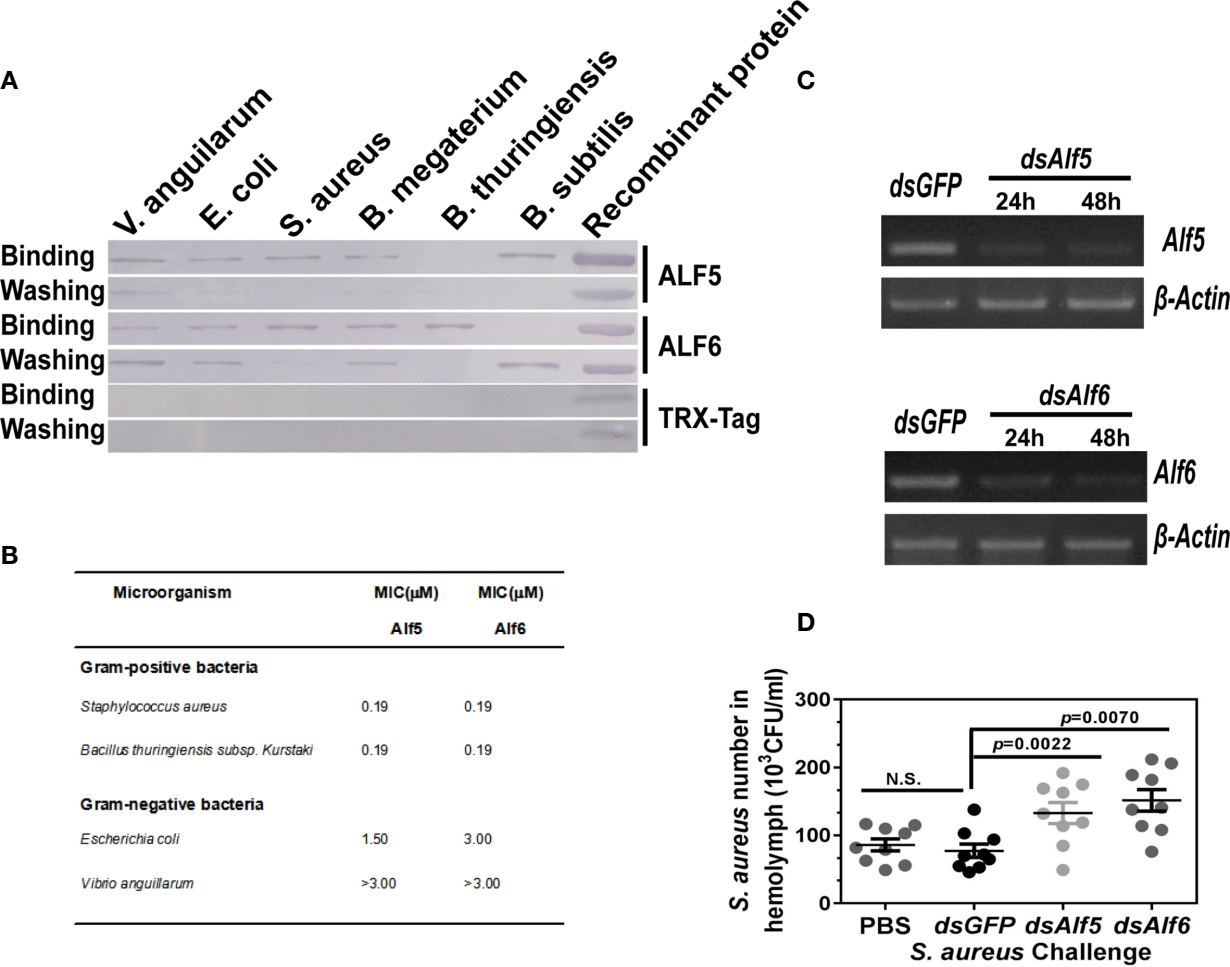
Functional investigation of ALF5 and ALF6. **(A)** Bacterial binding assay of recombinant protein of ALF5 and ALF6. Various bacterial strains (2 × 10^6^ cells) were incubated with purified recombinant protein of ALF5 and ALF6 (10 μg) After being washed with a 7% SDS and TBS solution, the bacterial pellets were collected and detected by Western blot with an anti-Histidine monoclonal antibody (1:4,000). The purified TRX protein was utilized as a negative control. **(B)** Antibacterial activity assay of rALFs. Bacterial cells (90 µl/well) were diluted to a final concentration with 2 × 10^5^ cfu/ml in Poor Broth, added into a 96-well microtiter plate. Double diluted rALF protein (10 µl/well) or the control protein (bovine serum albumin) were added to the 96-well plate. The original final concentration was 3 µM. The mixtures were incubated for 48 h with vigorous shaking at 30°C, and bacterial growth was evaluated by measuring the culture absorbance at 600 nm using a microplate reader. The minimal growth inhibition concentration (MIC) was expressed as the lowest final concentration of the protein at which no bacterial growth was observed compared with that in control. **(C)** Semi-quantitative RT-PCR detection of the knockdown efficiency in the *Alf*5- or *Alf6*-silenced shrimp. *β-Actin* was used as internal control. **(D)** The *in vivo* functional investigation of *Alf*5 and *Alf*6. After the expression of *Alf*5 and *Alf*6 were knocked down, bacteria (3 × 10^8^ CFU) were injected into shrimp (seven individuals in each group), 200 μl hemolymph was collected from each shrimp at 6 h post-injection, and bacterial numbers in 10 μl of hemolymph were counted after overnight cultivation. The PBS- and *dsGFP*-injected shrimps were regarded as control. Significant differences were analyzed by the student’s t-test and the p value are shown. N.S. means no significant differences.

To clarify whether the produced Alf5 and Alf6 are active AMPs in the antibacterial response of shrimp, their purified recombinant proteins (rAlf5 and rAlf6) were prepared and their binding activities to several bacterial strains were detected first. rAlf5 could bind to *V. anguillarum*, *E. coli*, *S. aureus*, *B. megaterium*, and *B. subtilis* but not to *B. thuringiensis*. rAlf6 showed the binding to all the tested bacteria, except for *B. subtilis*, while the control tag protein TRX could not bind to any of the bacteria (**Figure 5A**). Antimicrobial activities of recombinant Alfs were tested by liquid growth inhibition assays as minimum inhibitory concentration (MIC) values, rALF5 and rALF6 showed antimicrobial activities to the tested Gram-positive bacterial strains (*S. aureus* and *B. thuringiensis*) with MIC values at 0.19 mM, but showed no antimicrobial activities to the tested Gram-negative bacteria (*V. anguillarum* and *E. coli*) at the tested concentration (**Figure 5B**).

Next, the antibacterial activity of two *Alf*s was detected by an *in vivo* bacterial clearance assay. The shrimp were infected with *S. aureus* at 24 h post-*ds*RNA injection, the bacterial number in the control group and *Alf5*- or *Alf6*-silenced shrimp hemolymph were calculated and compared. **Figure 5C** shows that the expressions of *Alf5* and *Alf6* in shrimp were suppressed at the time detected (24 and 48 h post-*ds*RNA injection). In these conditions, a high number of *S. aureus* were found in both *Alf5*- and *Alf6*-silenced shrimp compared with those in the control group *(dsGFP*-injected shrimp), demonstrating that *Alf5* and *Alf6* were active in *S. aureus* clearance *in vivo* (**Figure 5D**).

### ECSIT is Needed for Signal Transmission From Toll3 to Dorsal

**Figure 6:**
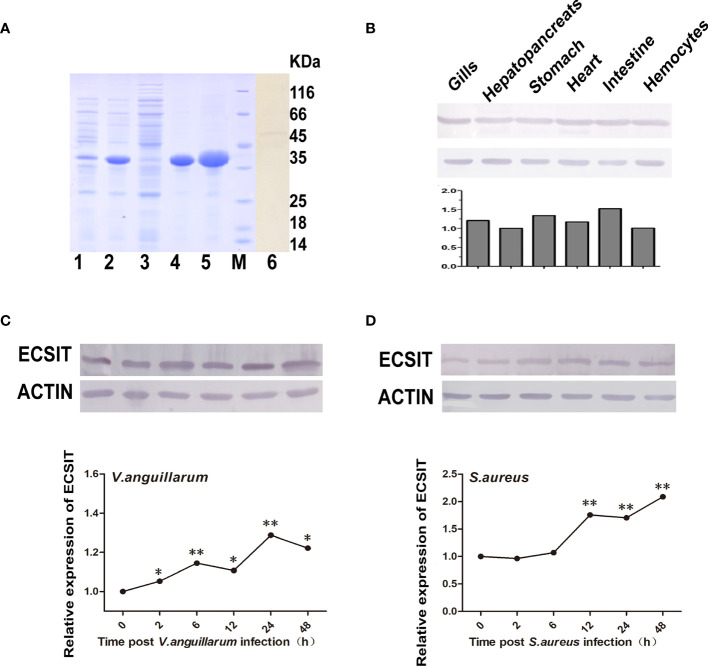
Recombinant expression, antibody detection, and expression pattern detection of ECSIT. **(A)** Recombinant expression, purification, and antibody detection of ECSIT. Lane 1, total protein of recombinant BL21 (DE3) cells without induction; lane 2, total protein of recombinant BL21 (DE3) cells after induction with 0.5 mM IPTG; lane 3, soluble protein fraction from the induced recombinant BL21 (DE3) cells; lane 4, insoluble protein fraction from the induced recombinant BL21 (DE3) cells; lane 5, purified recombinant protein of ECSIT domain; lane M, standard protein marker; lanes 6 and 7, Western blot detection of ECSIT in shrimp gills and intestine using an antibody against ECSIT. **(B)** Tissue distribution analysis of ECSIT in normal shrimp by Western blot, and **(b)** quantitative analysis of Western blot result in panel **(B)**. **(C)** ECSIT protein expression pattern in gills after *V. anguillarum* challenge by Western blot, and **(c)** quantitative analysis of panel **(C)**. **(D)** ECSIT protein expression pattern in gills after *S. aureus* challenge determined using Western blot, and **(d)** quantitative analysis of panel **(D)**. Significant differences were analyzed between the zero hour and other indicated time point samples by paired student’s *t*-test analysis, and they are indicated by asterisks (*p < 0.05, **p < 0.01). The experiments in **(B–D)** were performed three times, and similar results were obtained.

Firstly, the polyclonal antibody recognizing a specific single-band (~48 KDa) *ECSIT* protein in shrimp tissues was prepared by using purified recombinant protein of the ECSIT domain of the shrimp *ECSIT* gene (*MjEcist1*) (**Figure 6A**, line 6). The antibody could specifically recognize the purified recombinant ECSIT protein (~37 KDa) but not the control protein (recombinant ALF5 protein expressed and purified with the same system, **Supplementary Figure 1A**). The tissue distribution of ECSIT protein was studied by Western blot and was ubiquitously expressed in the six tested tissues, with high levels in the gill and digestive tract (**Figure 6B**). The expression of ECSIT in shrimp gills kept rising during Gram-negative bacteria (*V. anguillarum*) challenge (**Figure 6C**) and a higher level of ECSIT expression was seen after 6 h of challenge with *S. aureus* bacteria (**Figure 6D**). These results indicated that ECSIT protein participated in the antibacterial immunity of shrimp.

**Figure 7:**
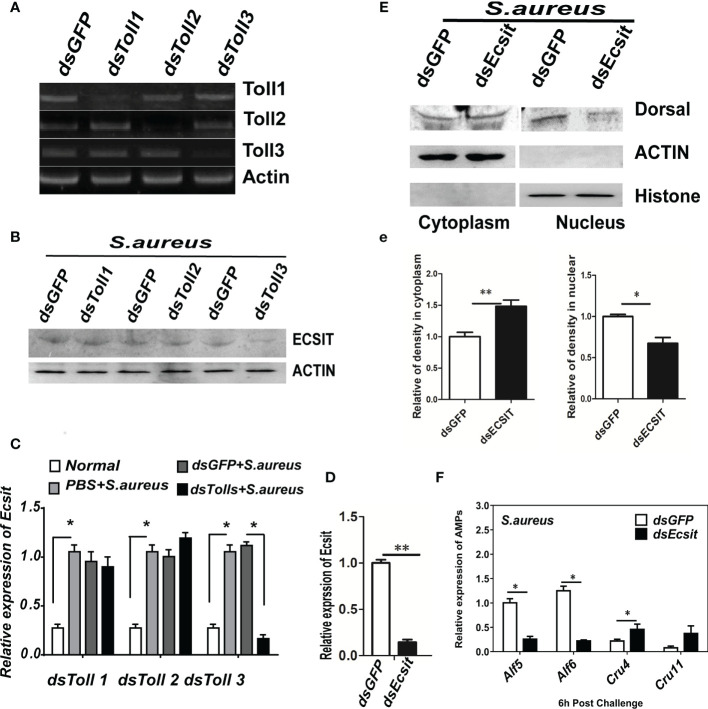
ECSIT is needed for signal transduction from Toll3 to Dorsal. **(A)** Detection of the knockdown efficiency of Tolls. The expression of Toll 1/2/3 in each Toll-silenced shrimp was detected by semi-quantitative PCR. *β*-ACTIN was used as the internal control. **(B)** ECSIT protein in Toll-silenced shrimp gills was detected by Western blot. A polyclonal antibody of ECSIT was used as primary antibody (1:500 dilution). **(C)** The transcription of the ECSIT gene in Toll-silenced shrimp gills was detected by qRT-PCR. β-Actin was used as the internal control. **(D)** Detection the knockdown efficiency of the ECSIT gene. *β*-Actin was used as the internal control. Western blot detection the nuclear translocation of Dorsal in ECSIT-silenced shrimp. (e) Statistical analysis of the results in **(E)**. **(F)** AMP expression levels in ECSIT-silenced shrimp gills at 6 h post-S. *aureus* infection were detected by qRT-PCR. All experiments were performed three times and similar results were obtained. Significant differences between samples were analyzed by paired student’s t-test and indicated by asterisks (*p < 0.05, **p < 0.01).

Subsequently, we detected whether the expression of ECSIT protein was affected by shrimp *Toll* knockdown during *S. aureus* infection. In this research, the specific knockdown of *Toll*s was confirmed and shown in **Figure 7A** before test the expression of ECSIT. In these conditions, an obvious reduction in ECSIT protein levels was seen in *Toll3*-silenced shrimp but not in the *Toll1*- or *Toll2*-silenced shrimp, suggesting that ECSIT is downstream of *Toll3* but not of the other two *Toll*s (**Figure 7B**). These results were consistent with *ECSIT* transcriptional levels in **Figure 7C**.

Then, we detected whether ECSIT functioned upstream of Dorsal. The cellular distribution of Dorsal and the AMP gene expression in *ECSIT*-silenced or control shrimp was detected 6 h after *ECSIT* gene knockdown and *S. aureus* infection. The nuclear translocation of Dorsal was inhibited in *ECSIT*-silenced and *S. aureus*-infected shrimp (**Figures 7D–e**). At the same time, the upregulated expression of two readout genes (*Alf*5 and *Alf*6*)* was also inhibited in *ECSIT*-silenced and *S. aureus-*infected shrimp (**Figure 7F**) but the expression of *Crustin11* was not affected, and that of *Crustin*4 was upregulated. These results were consistent with those in *Dorsal*-silenced shrimp, suggesting that they are functioned in the same signaling pathway. Taken together, these results strongly suggest that ECSIT was needed for signal transduction from Toll3 to Dorsal. A Toll3–ECSIT–Dorsal–Alf pathway may function in anti-*S. aureus* immunity in shrimp.

### The Toll3–Ecsit1–Dorsal–Alfs Axis Opposes *S. aureus* Infection in Shrimp

**Figure 8:**
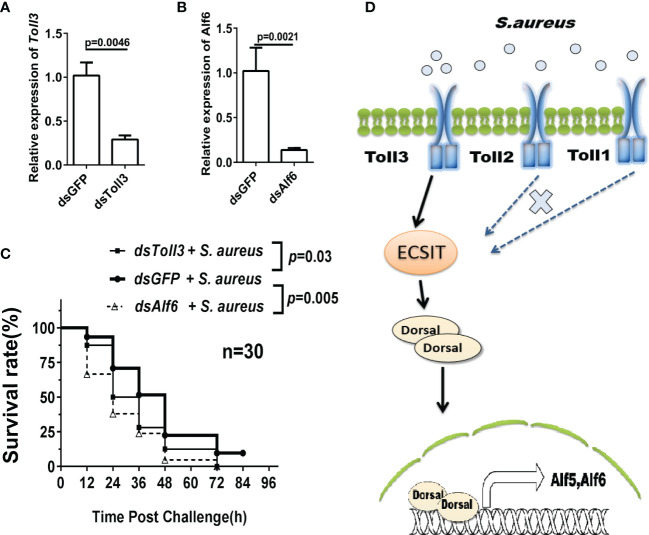
The Toll3–ECSIT–Dorsal–Alfs axis opposes *S. aureus* infection in shrimp. Survival rates of *Toll*3-RNAi and *Alf*6-RNAi shrimp infected with *S. aureus*. **(A, B)** Detection of the knockdown efficiency of *Toll*3 and *Alf*6. **(C)** Survival rate assay. Three groups were used, with 30 individuals in each group. Each shrimp was injected twice with 3 μg/g *ds*RNA for RNAi, the second injection was administered 24 h after the first injection. *GFP*-RNAi was utilized as the control. The *S. aureus* (3 × 10^8^ cells) challenge was performed after RNAi, and the number of dead shrimps was counted every 12 h.The data was analyzed by GraphPad Prism and the p value are showed. **(D)** Model for ECSIT intermediate Toll3–Dorsal–Alf pathway against bacterial infection.

To further verify whether the Toll3–ECSIT–Dorsal–Alf axis was functional in *in vivo* anti-*S. aureus* shrimp immunity, a survival assay was performed. Experimental shrimps were challenged with *S. aureus* at 24 h post-*dsToll3* or *dsAlf6* injection, and the shrimp survival rate was recorded every 12 h. **Figures 8A, B** show that the expression of *Toll3* and *Alf6* was effectively knocked down. The survival rates of the *Toll3*-silenced and *Alf6*-silenced shrimp were significantly lower than those of the control group (**Figure 8C**). These results, together with those above, suggested that the Toll3–Ecsit1–Dorsal–Alfs pathway plays an important role in the antibacterial immunity of shrimp (**Figure 8D**).

## Discussion

In the present study, the *in vivo* function of three Tolls in *M. japonicus* was investigated and showed different roles in anti-Gram-positive and anti-Gram-negative bacterial immune responses in shrimp. During *S. aureus* infection, the translocation into the nucleus of the NF-κB homologue *Dorsal* was induced, and the expression of several AMPs was induced in shrimp gills. *Ecsit* knockdown inhibited the nuclear translocation of Dorsal and expression of *Alf5* and *Alf6* genes. Furthermore, the expression of ECSIT protein and downstream signaling transduction were inhibited in *Toll3*-silenced shrimp but not in *Toll1*- or *Toll2*-silenced shrimp. In addition, Alf5 and Alf6 were active in binding to several bacterial strains and inhibiting the proliferation of *S. aureus in vivo*. The knockdown *Toll3* and *Alfs* led to remarkably decreased survival rates. Taken together, a Toll3–ECSIT–Dorsal–*Alf*s signal pathway active in kuruma shrimp under anti-*S. aureus* infection was disclosed. To our knowledge, this is the first report describing an antibacterial Toll signaling pathway in penaeid shrimp.

Database searching and sequence alignment analysis showed that the Toll3 in *M. japonicus* (*Mj*Toll3) had high sequence similarities with other crustacean Tolls (over 70% identity). However, most of the sequences that were submitted to the database were with no functional annotation, except the *Lv*Toll3 from *L. vannamei* shrimp (AEK86517.1, identity 97.2%) and *Pt*Toll3 from *Portunus trituberculatus* marine crab (AKV62617.1, identity 78%). The Toll3 proteins from those three species were ubiquitously expressed in all tested tissues and responded to the bacterial challenge. Overexpression of *Lv*Toll3 in *Drosophila* S2 cells showed that it was localized to the membrane and cytoplasm of cells, and its knockdown resulted in increased viral titer *in vivo* (35), indicating its role in recognizing viral infection, although *LvToll*4 was found to be more important (17). These results showed that some Tolls in shrimp may have a redundant function during infection with a given pathogen. This also applies to our findings, as knockdown each Toll led to significantly elevated *S. aureus* numbers *in vivo*, indicating they all functioned in anti-*S. aureus* infection. However, the knockdown *Toll1* and *Toll2* had no influence on the expression of ECSIT and the downstream signaling transduction (**Figure 6**), indicating that another ECSIT–Dorsal-independent pathway may exist. In *P. clarkia*, a Toll2–ATF4–ALF1/2 pathway was found to function in anti-*V. parahemolyticus* infection (30). Whether a similar pathway is involved in the anti-bacterial immunity of *M. japonicus* shrimp needs further exploration.

Besides that, phylogenetic analysis of shrimp *Toll* with *Toll*s from *Drosophila* and TLR from human showed that the shrimp *Toll3* clustered with *Toll6* from *D. melanogaster* (*DmToll*6, with 49% sequence identity), they were then clustered with *DmToll2*, *DmToll7*, and *DmToll8*. The human TLRs and *DmToll9* formed other independent branches (**Supplementary Figure 2**). In *Drosophila*, the Gal4/UAS system driving ubiquitous overexpression of *DmToll*6, *DmToll*7, and *DmToll*8 caused related phenotypic changes, namely, an abdominal closure defect, extra bristles, rough eyes, vein thickening, and lethality, indicating that these four genes have more conserved molecular structures and thus may regulate similar processes *in vivo*. *DmToll6* and *DmToll7* also function in cell migration targeting (36), embryonic development (37), neuronal networks (38–40), and olfactory development (41). It is worth noting that deletion mutant alleles of *DmToll6* and *DmToll7* were viable, fertile, and had no detectable defects in the septic-induced expression of antimicrobial peptide genes, suggesting that their overall innate immune response against bacteria does not have a severe defect (42, 43). These results were different from ours, since *MjToll*3 functioned in shrimp antibacterial immunity. Domain analysis of the shrimp and *Drosophila* Tolls showed that the LRR-CT domain is absent from the extracellular regions of *Dm*Toll*6*, and the TIR domain in the intracellular segment of most Tolls is missing from *Dm*Toll7 compared with *Mj*Toll3 (**Supplementary Figure 3**), which may be the reason for their lack of involvement in immune regulation. Moreover, the direct binding of Toll receptors to bacteria or PAMPs was reported in shrimp and mollusk animals (16, 44), showing that another activation mode for the Toll signaling pathway exists in invertebrates, perhaps another reason for their functional differences.

For aquatic invertebrates, the gills, together with the digestive tract, are the organs that directly face environmental pathogens. Besides that, hemocytes are considered as the direct immune response operator in animals (16, 17). Shrimp *Tolls* are widely distributed genes with high transcription in these tissues (15, 17). In our research, bacterial challenge led to rapid and continuous upregulation of three Toll genes in gills and intestinal tissues (**Figure 1A** and **Supplementary Figure 4**). The nuclear translocation of Dorsal in gills was suppressed only in *Toll*3- or *ECSIT*-silenced shrimp but not in *Toll*1- or *Toll*2-silenced shrimp (**Figure 5**). However, Sun et al. observed that the induced expression of Tolls by bacterial challenge in hemocytes was transient and occurred later (12 or 24 h post-infection). Moreover, the nuclear translocation of Dorsal in shrimp hemocytes was suppressed not only in *Toll*3-silenced shrimp but also in *Toll*1- or *Toll*2-silenced shrimp (16). Correspondingly, the AMPs regulated by Toll receptors upon bacterial challenge in hemocytes also differed from those in our research on gills. These results showed that the activation, signal transduction, and effector gene expression of the Toll pathway in those two shrimp tissues are discrepant, and the response in gills is more subtle than that in hemocytes. A similar phenomenon was also found in fruit fly. Genome sequencing showed that the expression of AMPs in *Drosophila* follows a complex pattern with tissue or temporal differences and is specific for each peptide. The regulatory mechanism of local (epithelial cells from the tracts that faced the microorganisms directly) or systemic induction of AMPs was different (45). For example, the expression of the *Drosomysin* gene was regulated not only by the Toll pathway in the fat body during the systemic response, but also by the *Imd* pathway in the trachea as local response (46).

To date, there are only a few reports on the function of *ECSIT* gene in invertebrates, namely, two shrimp *ECSITs* from *M. japonicus* and *Exopalaemon carinicauda* (26, 47), one mollusk *ECSIT* from *Crassostrea hongkongensis* (48), one mud crab *ECSIT* from *Scylla paramamosain*, and one *Drosophila ECSIT* (32, 49). Our previous research and the data in this article showed that shrimp *ECSIT* was necessary for antibacterial signal transduction from *Mj*Toll3 to Dorsal. In mammalian cells, ECSIT plays a key role in the TLR4 signaling pathway. A complex of TAK-1–ECSIT–TRAF6 was needed for the activation of NF-κB, the interaction of TRAF6 with ECSIT, leading to the ubiquitination of ECSIT at lysine (K) 372 residue, results in the interaction of P50/P65 NF-κB proteins with ubiquitinated ECSIT in the nucleus, which was necessary for the production of proinflammatory cytokines and affecting gene expression in response to TLR4 stimulation (50, 51). Additionally, ECSIT was also located in the mitochondrial complex I, and implicated in complex stability and mitochondrial and cellular reactive oxygen species production during bacterial infection, thus contributing to the bactericidal activity of macrophages (52). In arthropods, the interaction of TRAF6 with ECSIT and their role in regulating the expression of AMPs are reported in *Drosophila* and mud crab (32, 49). However, the functional connection of ECSIT and Toll receptors is still unclear. In our research, ECSIT was proved to be needed for signal transduction from Toll3 receptor to Dorsal during *S. aureus* infection, and the ubiquitination and interaction of ECSIT with TRAF6 in *M. japonicus* was also discovered (data not shown). However, the ubiquitination site lysine (K) 372 residue in mammalian ECSIT is not conserved in shrimp ECSIT. Whether shrimp ECSIT functioned as the same way in mammalian system to activate Dorsal translocation into the nucleus in shrimp still needs further exploration.

In conclusion, a novel Toll3–ECSIT–Dorsal–Alf signal pathway was identified in kuruma shrimp, and this finding enables a systematic understanding of the Toll signaling pathway in shrimp immunity. This study also provides deep insights into and enhanced comparison of the Toll/TLR signaling pathway in various species.

## Data Availability Statement

The original contributions presented in the study are included in the article/**Supplementary Material**. Further inquiries can be directed to the corresponding author.

## Ethics Statement

All animal operations in this study were approved by the Animal Care and Welfare Committee at the Shandong University School of Life Sciences (SYDWLL-2021-99), and all efforts were made to minimize suffering.

## Author Contributions

Conceived and designed the experiments: C-jK. Performed the experiments: DD, X-jS, MY, LG, and QC. Analyzed the data and wrote the paper: C-jK and X-jS. All authors contributed to the article and approved the submitted version.

## Funding

The current study was supported by the National Key R&D Program of China (No. 2018YFD0900303) and the National Natural Science Foundation of China (Grant Nos. 31572655 and 31272689).

## Conflict of Interest

The authors declare that the research was conducted in the absence of any commercial or financial relationships that could be construed as a potential conflict of interest.

## Publisher’s Note

All claims expressed in this article are solely those of the authors and do not necessarily represent those of their affiliated organizations, or those of the publisher, the editors and the reviewers. Any product that may be evaluated in this article, or claim that may be made by its manufacturer, is not guaranteed or endorsed by the publisher.
