# Supplementary material for: The ECSIT Mediated Toll3-Dorsal-ALFs Pathway Inhibits Bacterial Amplification in Kuruma Shrimp
Source: Front Immunol. 2022 Jan 31;13:807326. doi: 10.3389/fimmu.2022.807326 (PMC8841768; doi:10.3389/fimmu.2022.807326)

Supplementary Material

1. Supplement figure legends

Supplement Figure 1. Purified protein and antibody detect of Dorsal,ECSIT and ALF5 . **(A)** Detect the protein and antibody of Dorsal. M, standard protein marker; Lane1, total protein of recombinant BL21(DE3) cells without induction; lane 2, total protein of recombinant BL21(DE3) cell after induced with 0.5 mM IPTG; lane 3;western blot detection of recombinant expressed Dorsal protein using antibody against Dorsal (50KDa) **(B)** Detect the protein and antibody of ECSIT and ALF5. M, standard protein marker; Lane1, total protein of recombinant BL21(DE3) cells without induction; lane 2, total protein of recombinant BL21(DE3) cell after induced with 0.5 mM IPTG; lane 3, purified recombinant protein of ECSIT protein; lane 4,Western blot detection of recombinant expressed rALF5 using antibody against ECSIT(37KDa); land 5,Western blot detection of recombinant expressed ECSIT protein using antibody against ECSIT (37KDa); lane 6, purified recombinant expressed ALF5 protein (35KDa).

Supplement Figure 2. Phylogenetic analysis of Tolls/TLRs from shrimp, fruit fly, and human. The neighbor-joining tree was constructed by MEGA 5.0 with 1000 bootstrap. *Mj*Toll1, *Mj*Toll2, and *Mj*Toll3 are labeled with solid triangles.

***M.Japonicus* Toll1**

***M.Japonicus* Toll2**

***D.melanogaster***

***D.melanogaster***

***D.melanogaster***

***D.melanogaster***

***D.melanogaster***

***D.melanogaster***

***D.melanogaster***

***D.melanogaster***

***M.Japonicus* Toll3**

***H.sapiens***

***H.sapiens***

***H.sapiens***

***H.sapiens***

***H.sapiens***

***H.sapiens***

***H.sapiens***

***H.sapiens***

***H.sapiens***

***H.sapiens***

***D.melanogaster***

Supplement Figure 3. Schematic representations of the domain topology of Toll/TLR from shrimp (*Mj*Toll1-3), human (*Hs*TLR1-9), and fruit fly (*Dm*Toll1-9). A simple modular architecture research tool (SMART, http://smart.embl-heidelberg.de) was used to analyze the protein domain topology.

Supplement Figure 4. Expression patterns of Tolls in shrimp intestine. The expression pattern of Tolls in shrimp intestine upon *V. anguillarum* or *S. aureus* infection was detected by Q-PCR **(A-C)**. The significant differences were analyzed between the bacteria-challenged samples and the PBS-injected samples by paired t-test analysis and were marked by asterisks (**p* < 0.05, ***p* < 0.01).

1. Supplement Figures

Fig.1


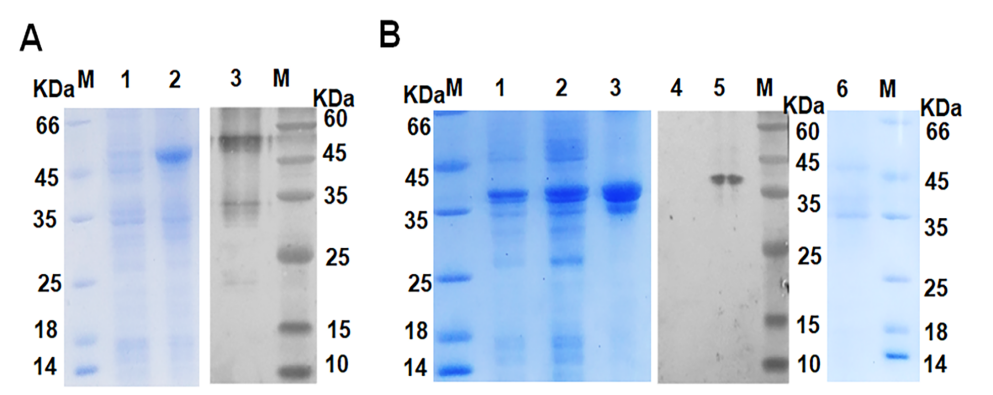


Fig.2


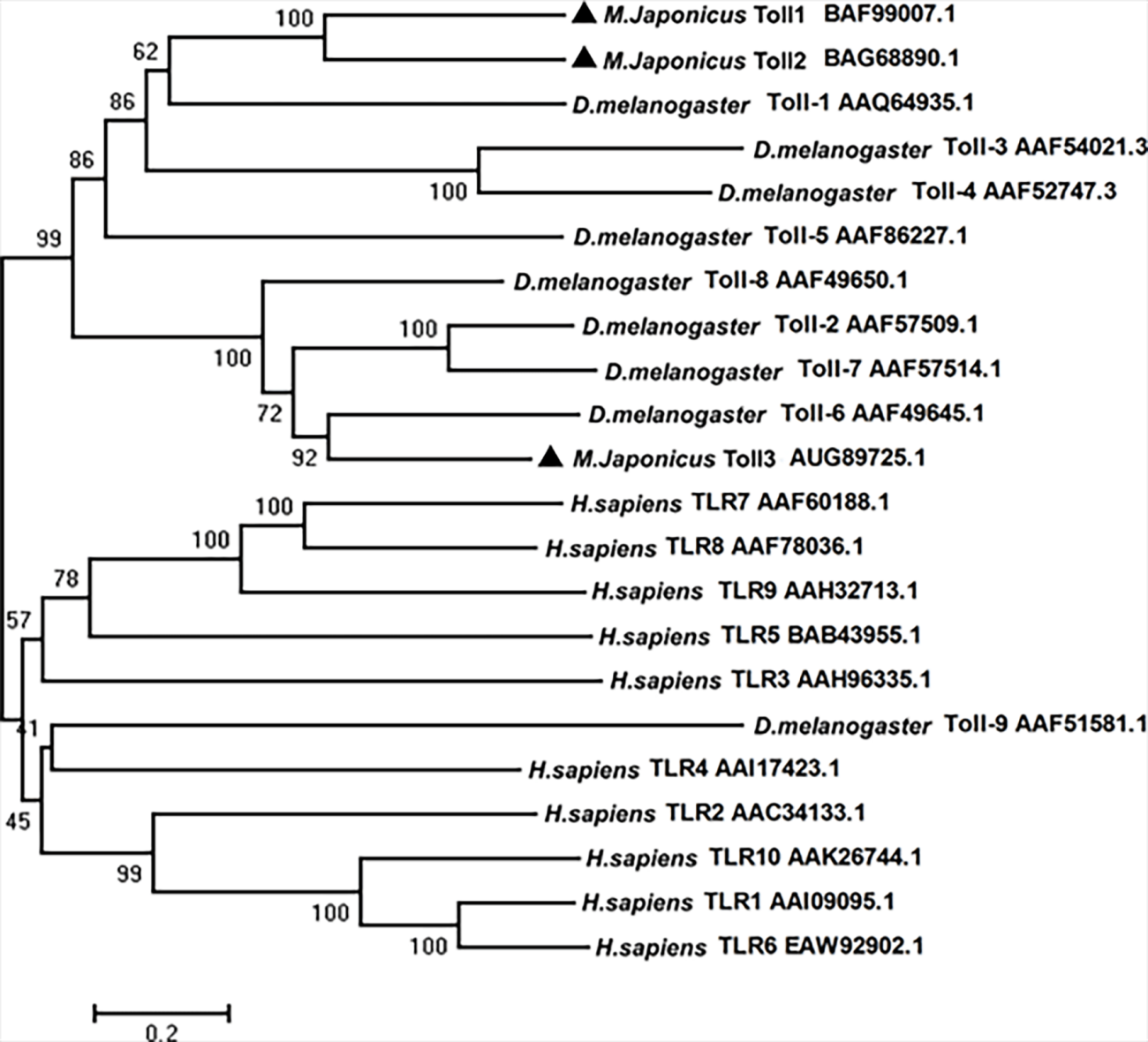


Fig.3


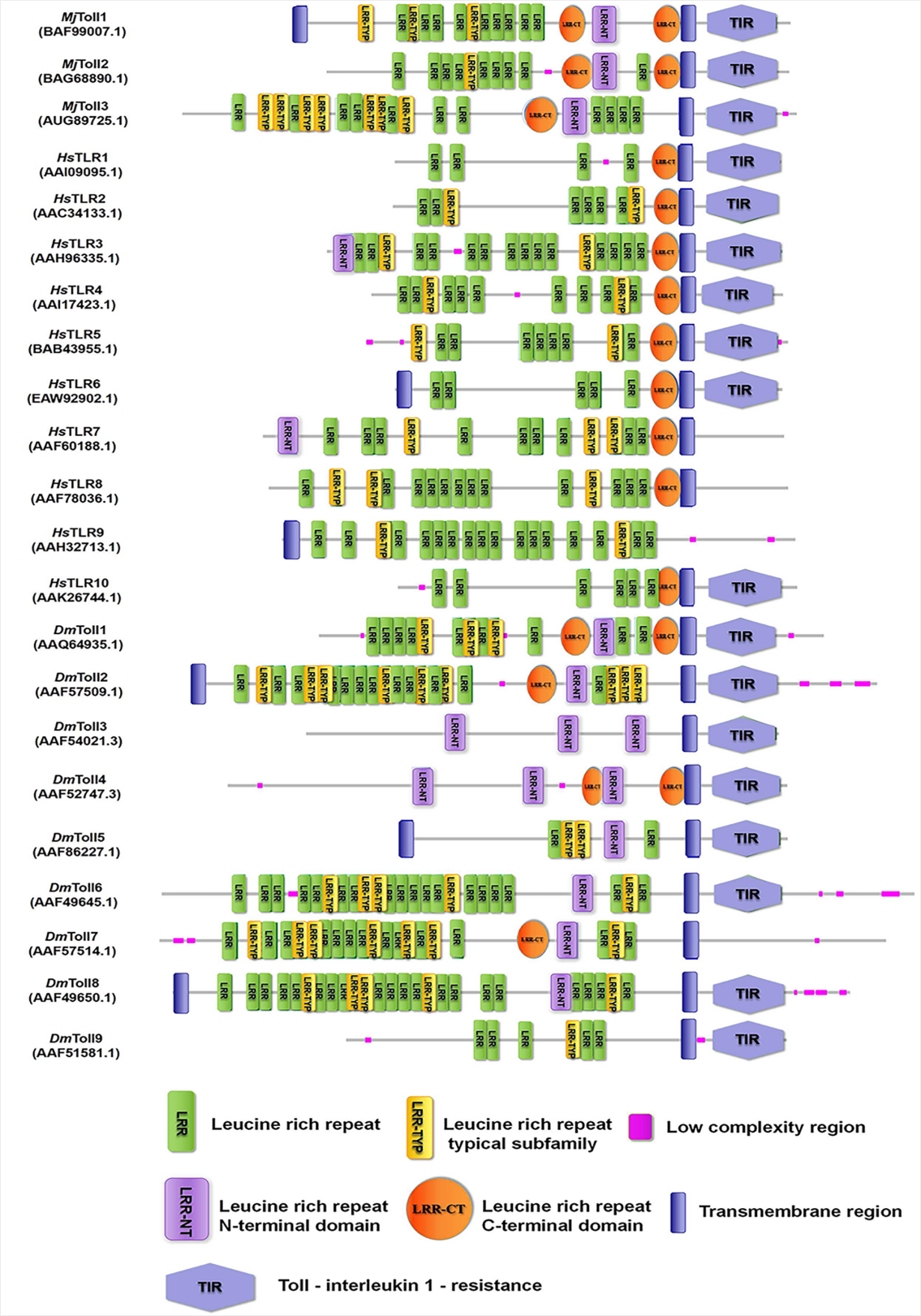


Fig.4


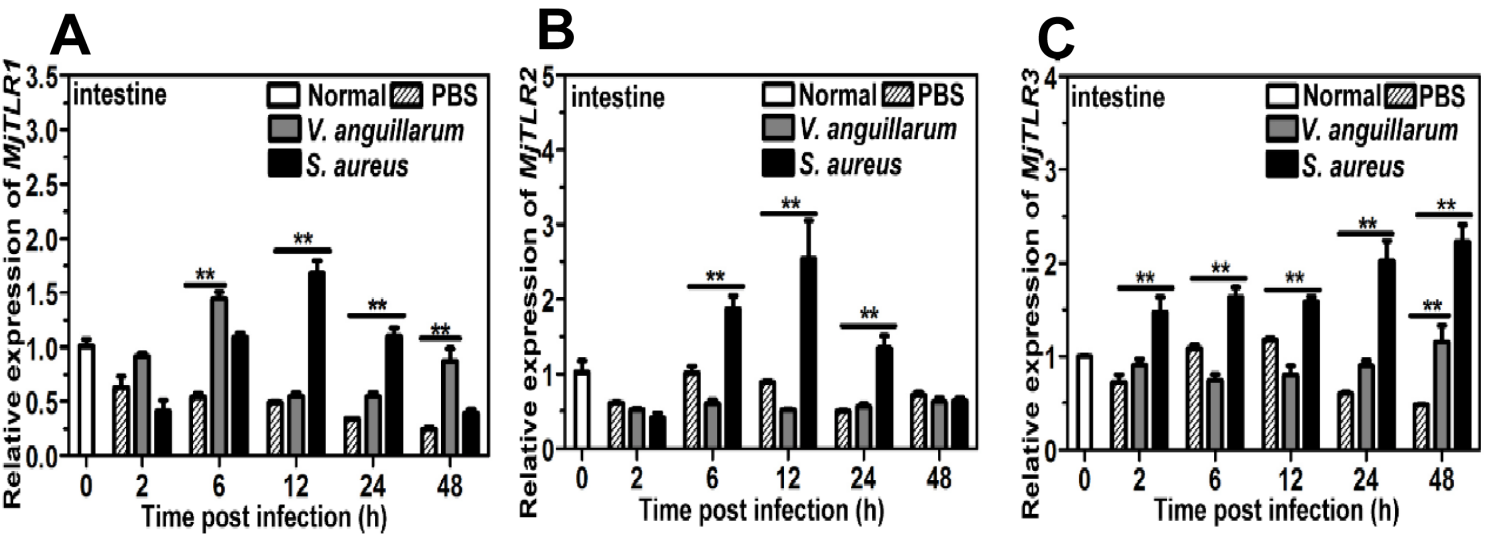

Supplement: Supplementary file 1 [file DataSheet_1.docx]
